# Disentangling the mechanisms regulating nighttime transpiration during drought across plant life forms

**DOI:** 10.1093/plphys/kiaf331

**Published:** 2025-07-28

**Authors:** Laura Fernández-de-Uña

**Affiliations:** Assistant Features Editor, Plant Physiology, American Society of Plant Biologists; Department of Plant Biology and Soil Sciences, Universidade de Vigo, 32004 Ourense, Spain

Transpiration is the process through which plants transport water from the roots to the leaves, where it diffuses into the atmosphere through the stomata. Plants regulate the exchange of water and CO_2_ between the plant and the atmosphere largely through stomatal aperture. When stomata are open, they allow the uptake of CO_2_, needed for photosynthesis, as water is released to the atmosphere. Transpiration also delivers water and nutrients to the plant, needed for tissue hydration and photosynthetic and other chemical reactions, and it provides leaf cooling, among other functions. Because there is no carbon gain and leaf cooling needs are generally low or absent at night, stomata have been traditionally assumed to close in the absence of solar radiation, and nocturnal transpiration has been considered negligible. Indeed, diel sap flow measurements often assumed that transpiration reaches 0 at some point every night and nocturnal transpiration occurs rarely under specific environmental conditions (Granier 1987). However, nighttime transpiration has been proven to be substantial across plant life forms (Caird et al. 2007; Dawson et al. 2007). Nighttime transpiration may serve several functions, including the release of CO_2_ produced in respiration, nutrient and/or oxygen uptake, restoration of water reserves and water flow in cavitated xylem conduits, or leaf cooling during warm nights (Wang et al. 2021). Wang et al. (2021) proposed that plants may transpire at night as long as the benefits outweighed the costs, finding that nighttime diffusive conductance—a function of transpiration and vapor pressure deficit—decreased as drought increased in birch.

Stomatal aperture depends on the position of the two stomatal guard cells enclosing the stomatal pore (Figure), which in turn depends on cell volume and turgor. Two main mechanisms have been proposed to control stomatal closure under drought (Buckley 2019). On one hand, guard cell turgor may decrease as plant water potential drops under drought (hydropassive regulation). On the other, the synthesis of the hormone abscisic acid (ABA) may induce the loss of solutes from guard cells, in turn reducing their turgor and prompting stomatal closure (Buckley 2019). What mechanism prevails in regulating nighttime transpiration remains largely unknown.

**Figure.**
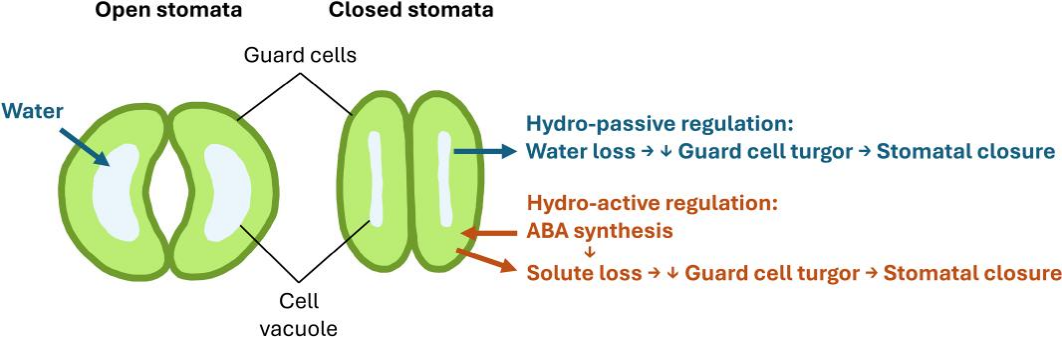
Mechanisms regulating stomatal closure in vascular plants.

In a recent article in *Plant Physiology*, Pereira et al. (2025) explored the potential mechanisms regulating nighttime transpiration during drought in terrestrial vascular plants presenting contrasting stomatal regulation strategies under drought: a fern (*Cyrtomium falcatum*) that closes stomata hydropassively; a gymnosperm (*Pinus taeda*) in which stomata are regulated by ABA; a woody angiosperm species (*Umbellularia californica*) that regulates stomatal closure using ABA in the earlier stages of drought and hydropassively as drought progresses; and two varieties of a herbaceous angiosperm (*Solanum lycopersicum*), a wild type that relies on ABA for stomatal closure and a mutant unable to synthesize ABA.

In all species, daytime and nighttime transpiration rates remained constant as drought progressed until a species-specific soil moisture level was reached, after which both types of transpiration progressively decreased (Pereira et al. 2025). The only exception was the *S. lycopersicum* mutant, for which transpiration rates remained constant across drought levels, during the day and at night. In the fern and the conifer, nocturnal transpiration started to decline at greater soil water deficits than daytime transpiration, while in the two angiosperm species the decline started at similar drought levels for both variables. All species, with the exception of the *S. lycopersicum* mutant, showed an average increase of 10% to 13% in the ratio between night- and daytime transpiration when soil transpirable water was significantly reduced, with nocturnal transpiration representing up to 50% of daytime transpiration in *U. californica* and 90% in *S. lycopersicum*. Therefore, nocturnal transpiration cannot be considered a constant, negligible variable across plant life forms.

ABA concentrations were higher in the two angiosperm species than in the gymnosperm and, to a much greater extent, the fern, even under nondrought conditions (Pereira et al. 2025). In the fern, nighttime transpiration decreased as leaf water potential dropped, while foliar ABA concentrations remained minimal. Conversely, in the two angiosperms, the decrease in nighttime transpiration was associated to a significant increase in leaf ABA concentrations as water potentials dropped, suggesting a strong hormonal regulation of stomatal closure. This was particularly the case for the herbaceous angiosperm, for which ABA levels continued rising along the drought, reaching the highest values of all the studied species, while the mutant type, unable to synthesize ABA, did not alter day- or nighttime transpiration during drought. In the woody angiosperm *U. californica*, however, ABA dynamics with dropping leaf water potential were nonlinear, decreasing at higher levels of drought stress and thus switching to a hydropassive regulation of stomatal aperture (Pereira et al. 2025). Conversely, in pine, ABA increased only under severe drought, after nighttime transpiration had started to decline, suggesting a switch from hydropassive regulation under mild water stress to ABA regulation under severe water deficits (Pereira et al. 2025). Therefore, the mechanisms regulating stomatal closure at night are species specific and consistent with those regulating stomatal closure during the day in each studied species.

Pereira et al. (2025) show that nocturnal transpiration represents an important part of daily transpiration in all plant life forms, particularly under drought as daytime transpiration decreases. It also shows that nighttime transpiration is not constant but decreases as drought progresses. Previous work showed that nighttime transpiration did not exacerbate drought stress in grapevine, partly due to the much lower proportion of nighttime transpiration as compared with daytime water losses (Dayer et al. 2021). However, in light of the results obtained by Pereira et al., this may be due to the concurrent reduction in nighttime transpiration as drought progresses. Given the projected increase in nocturnal temperatures, future research should estimate, for a broader number of species and ecosystems, how nighttime transpiration affects plant performance under drought, especially in terms of water use efficiency.

## Data Availability

No new data were generated or analysed in support of this article.
